# Sirtuins promote brain homeostasis, preventing Alzheimer’s disease through targeting neuroinflammation

**DOI:** 10.3389/fphys.2022.962769

**Published:** 2022-08-15

**Authors:** Mateusz Watroba, Dariusz Szukiewicz

**Affiliations:** Department of Biophysics and Human Physiology, Faculty of Health Sciences, Medical University of Warsaw, Warsaw, Poland

**Keywords:** sirtuins (SIRTs), brain homeostasis, neuroinf lammation, alzheimer’s disease, aging

## Abstract

Both basic pathomechanisms underlying Alzheimer’s disease and some premises for stipulating a possible preventive role of some sirtuins, especially SIRT1 and SIRT3, protective against Alzheimer’s disease-related pathology, are discussed in this article. Sirtuins can inhibit some processes that underlie Alzheimer’s disease-related molecular pathology (e.g., neuroinflammation, neuroinflammation-related oxidative stress, A*β* aggregate deposition, and neurofibrillary tangle formation), thus preventing many of those pathologic alterations at relatively early stages of their development. Subsequently, the authors discuss in details which mechanisms of sirtuin action may prevent the development of Alzheimer’s disease, thus promoting brain homeostasis in the course of aging. In addition, a rationale for boosting sirtuin activity, both with allosteric activators and with NAD^+^ precursors, has been presented.

## Introduction

Alzheimer’s disease (AD) is a neurodegenerative disorder, clinically manifesting with a progressive loss of memory and cognitive functions (Scheltens et al., 2016; Fernando and Wijayasinghe, 2021). Histopathologic findings in AD patients’ brains usually occur much earlier than clinical manifestations of the disease; they include *β*-amyloid deposition in the interneural space and accumulation of abnormal, hyperphosphorylated tau proteins within neurons (Sperling et al., 2011; Kumar et al., 2015; Hanseeuw et al., 2019).

## Review

### General pathomechanisms of Alzheimer’s disease

*β*-amyloid deposits, referred to as A*β* aggregates, are produced during degradation of amyloid-precursor protein (APP) which is quite large transmembrane glycoprotein, cleaved by *β*- and *γ*-secretases to about 40-aminoacid peptides called A*β* monomers (Zheng and Koo, 2011; Chen et al., 2017). APP protein itself is quite abundant in the brain, playing a signaling role in neuronal development, maintenance of synapses, and neuronal homeostasis (van der Kant and Goldstein, 2015). Some A*β* monomers tend to condensate into insoluble oligomers, in the form of fibrils or plaques. Senile plaques, very characteristic for AD, contain mainly fibrillary proteins referred to as A*β*_1-42_. A*β* neurotoxicity is attributed mainly to its soluble oligomeric form, which is capable to disrupt intraneuronal calcium homeostasis through causing an excessive calcium influx into the neurons, with a subsequent mitochondrial damage and neuronal death (Arispe et al., 1993; Zhao et al., 2012; Colvin et al., 2016; Wälti et al., 2016) (Supplementary Data Sheet S1). In some mouse models of AD, progressive deposition of A*β* aggregates is found mainly in hippocampus and cerebral cortex (Zhao et al., 2012). Intraneuronal deposition of abnormal tau proteins can usually be observed within a few years after the onset of A*β* deposition in the interneural space (Musiek and Holtzman, 2015; Sasaguri et al., 2017). Tau protein itself is a microtubule-associated protein, stabilizing the microtubules and thus significant for axonal transport. Abnormal phosphorylation of tau proteins results in their dissociation from microtubules and formation of fibrillary structures called neurofibrillary tangles (NFTs) (Iqbal et al., 2010; Wang and Mandelkow, 2016). Intraneuronal accumulation of NFTs results in neuronal malfunction, followed by neuronal death.

In addition, abnormalities in cerebral metabolism of cholesterol have been found in Alzheimer’s disease (Feringa and van der Kant, 2021). Accumulation of cholesterol within neurons promotes APP interactions with *β*- and *γ*-secretases, resulting in the production of aforementioned A*β* aggregates (Di Paolo and Kim, 2011). Because apolipoprotein E regulates cholesterol transport to the brain and lipid rafts function in astrocytes, a positive correlation can be found between AD risk and possessing certain alleles of apolipoprotein E-encoding gene (Corder et al., 1993; Wang et al., 2021).

### Neuroinflammation and its role in the pathogenesis of AD

In addition to accumulation of abnormal protein aggregates, neuroinflammation—i.e., inflammation within the central nervous system (CNS) also plays a role in the pathogenesis of AD (Heneka et al., 2015; Heppner et al., 2015; Calsolaro and Edison, 2016; Hampel et al., 2020). Neuroinflammatory response is an element of innate immunity, dependent on many types of cells, including microglial cells, astrocytes, cerebral vascular endothelial cells, mast cells and leukocytes reaching the cerebrospinal fluid through abnormally permeable blood-brain barrier (Das Sarma, 2014; 't Hart and den Dunnen, 2013). However, in the further part of this work, we focus mainly on microglial cells and astrocytes (Colombo and Farina, 2016; Ransohoff, 2016). The problem with neuroinflammation is that it can be potentially neurotoxic in its chronic form, despite being useful and neuroprotective in its acute form, through removal of pathogens from the brain (Kinney et al., 2018). Pro-inflammatory activity of microglial cells tends to increase with age, resulting in the increased production of pro-inflammatory mediators, inducing neuroinflammation, and increased permeability of blood-brain barrier (Schuitemaker et al., 2012; Elahy et al., 2015). Microglial cells obtained from old people show abnormalities in their morphology and function, impairing phagocytosis, proteostasis, and cell capability for migration (Mosher and Wyss-Coray, 2014). Furthermore, neuroinflammation can be additionally aggravated by the presence of abnormal protein aggregates, such as A*β* aggregates (Zenaro et al., 2017). In AD patients, a positive correlation is observed between the abundance of A*β* aggregates and intraneuronal deposits of tau proteins, and the extent of pro-inflammatory phenotype induction in microglial cells and blood-brain barrier permeability (Parbo et al., 2017; Dani et al., 2018; Nordengen et al., 2019). Microglial cells which have transited from their homeostatic phenotype to pro-inflammatory phenotype are located mainly in the vicinity of senile plaques (Heneka et al., 2015; Navarro et al., 2018). A*β* aggregates are responsible for such phenotypic transition of microglial cells, which results in the induction of many pro-inflammatory mediators promoting neuronal deaths, such as IL-1*β*, IL-6, TNF-*α*, chemokines, nitric oxide and prostaglandins (Glass et al., 2010). In addition, it is supposed that neuroinflammation can promote NFT formation in AD patients (Kitazawa et al., 2004; Kinney et al., 2018). Moreover, elevated concentrations of circulating pro-inflammatory cytokines (IL-1, IL-6, TNF-*α*) have been found in people suffering from dementia (Koyama et al., 2013; Lai et al., 2017; Darweesh et al., 2018; Shen et al., 2019).

Microglial cells comprise a component of innate immunity and are basically derived from macrophages (Ginhoux et al., 2010). Main functions of their homeostatic, phenotypically quiescent forms in healthy brain include elimination of pathogens, repairing tissue damage, immune surveillance, and homeostatic functions (maintenance of neurogenesis, neuronal plasticity, and synaptic well-being, thus promoting proper cognitive skills) (Nimmerjahn et al., 2005; Ji et al., 2013; Parkhurst et al., 2013). Phenotype of microglial cells can be switched from homeostatic to pro-inflammatory by pathogen-associated molecular patterns (PAMPs) and damage-associated molecular patterns (DAMPs), such as lipopolysaccharides of bacterial walls (LPS), misfolded proteins, or even some pesticides and air pollutants (Lull and Block, 2010). Such phenotypically switched microglial cells change their morphology, as well as activate phagocytosis and inflammation-associated signaling pathways (ElAli and Rivest, 2016; Minter et al., 2016; Salter and Stevens, 2017). In addition, the extent of this kind of phenotypic alteration of microglial cells increases with age, along with other innate immunity associated pro-inflammatory phenomena, such as toll-like receptor (TLR) signaling and inflammasome activation (Sierra et al., 2007; Cribbs et al., 2012). Several kinds of receptors mediate microglial cell phenotypic transition from homeostatic to pro-inflammatory, including TLRs, nucleotide-binding oligomerization domain-like receptors (NLRs), receptors for advanced glycation products, formyl peptide receptors, scavenger receptors, and receptors for immunoglobulin Fc fragments and complement components (Doens and Fernández, 2014; Fiebich et al., 2018). In the course of Alzheimer’s disease, microglial cells interact with A*β* aggregates through such receptors as TLR2, TLR4, TLR6, TLR9, scavenger receptors such as CD36, CD37, and scavenger receptor A1 (SR-A1), as well as receptors for advanced glycation products and complement components, like complement receptor 3 (CR3) (Doens and Fernández, 2014; Fiebich et al., 2018). In the course of aging, as well as in metabolic syndrome-associated systemic inflammation, microglial cells can be abnormally recruited to induce neuroinflammation (Perry and Teeling, 2013; Niraula et al., 2017). Both metabolic syndrome-associated systemic inflammation and aging-associated systemic inflammation are characterized by increased plasma concentrations of IL-6 and TNF-*α*, which is positively correlated with cognitive impairment and AD-resembling symptoms. This may suggest that such symptoms are mediated by phenotypic switching of microglial cells from their homeostatic/surveillant phenotype to pro-inflammatory phenotype, due to increased systemic concentration of pro-inflammatory cytokines (Holmes et al., 2011).

Studies on mouse models of AD indicate that LPS-stimulated microglial cells produce increased amounts of IL-1*β* which in turn stimulates astrocytes to produce chemokines, such as chemokine C-C motif ligand 2 (CCL2), chemokine C-X-C motif ligand 1 (CXCL1) and chemokine C-X-C motif ligand 10 (CXCL10) (Lopez-Rodriguez et al., 2021). Results of those studies suggest that microglia-astrocyte interactions in response to microglial cell acquiring a pro-inflammatory phenotype may aggravate neuroinflammation, which can sometimes result in the impairment of cognitive functions (González-Reyes et al., 2017). Microglial cells which have acquired a pro-inflammatory phenotype in A*β*-dependent manner may adhere to the sites of A*β* deposition as disease-associated microglia (DAM) (Keren-Shaul et al., 2017; Shahidehpour et al., 2021). In early stages of AD, DAM cells can be useful, removing A*β* aggregates in triggering receptor expressed on myeloid cells 2 (TREM2)-dependent manner (Keren-Shaul et al., 2017; Ulland and Colonna, 2018). Possessing some rarely occurring alleles of TREM2 encoding gene is a risk factor of late-onset Alzheimer’s disease (Gratuze et al., 2018). In the course of aging, replicative stress imposed on microglial cells can hinder their efficacy in A*β* clearance, which may promote A*β* deposits growing larger (Hu et al., 2021). This may in turn functionally overload microglial cells, decreasing the effectiveness of phagocytosis because of reduced expression of A*β*-binding proteins, such as SR-A1, CD36 and receptor for advanced glycation products (RAGE), as well as reduced expression of A*β*-degrading enzymes (Hickman et al., 2008). Overaccumulation of A*β* aggregates in DAM cells may also promote neuroinflammation through stimulating the expression of pro-inflammatory mediators (IL-1, IL-6, TNF-alfa) as well as other neurotoxic substances that can promote the progress of AD (e.g., nitric oxide and superoxide anion) (Block et al., 2007; Hickman et al., 2008; Smith et al., 2012; Deczkowska et al., 2018). Furthermore, intracellular accumulation of A*β* aggregates may result in microglial cell necrosis, with a subsequent release of A*β* aggregates back to the extracellular space, which can further promote A*β* deposits enlargement (Baik et al., 2016). Phagocytic efficacy of microglial cells can be restored by reducing A*β* burden (Krabbe et al., 2013). Since chronic and excessive imposing of pro-inflammatory phenotype on microglia promotes formation of neurofibrillary tangles (intraneuronal deposits of tau proteins), moderating this kind of microglial cell phenotypic transition is considered to be a potentially useful strategy in the prevention and treatment of Alzheimer’s disease (Kitazawa et al., 2004).

### Pro-inflammatory signaling pathways within microglial cells

A*β* binding to TLR receptors on microglial cells activates the same signaling pathways as are generally used for pathogen destruction. Directly, it can activate myeloid differentiation primary response (Myd88) transcription factor, which can transactivate other transcription factors, including nuclear factor kappa B (NF-κB) (Kawai and Akira, 2007). Active NF-κB may in turn promote the production of pro-IL-1*β* and NLR family pyrin domain containing 3 (NLRP3) cytoplasmic receptor (Bauernfeind et al., 2009). IL-1*β* is the main pro-inflammatory cytokine associated with neuroinflammation in the course of Alzheimer’s disease (Shaftel et al., 2008). In addition, it has been found that increased IL-1*β* expression in human microglial cells in the course of aging is underlied by a selective hypomethylation of IL-1*β* gene proximal promoter (Cho et al., 2015). However, IL-1*β* is synthesized in the form of inactive precursor—pro-IL-1*β* which can be transformed to IL-1*β* in the presence of active caspase 1, an intracellular pro-inflammatory caspase (Halle et al., 2008). Caspase 1 is also produced in the form of its inactive precursor—procaspase 1, and transforming of procaspase 1 to caspase 1 requires its proteolytic processing in inflammasomes, the most important being NLRP3 inflammasome. NLRP3 inflammasome consists of NLRP3 receptor, apoptosis-associated speck-like protein containing a CARD (ASC protein), and caspase 1 protease. Inflammasomes are responsible for detection of potential tissue insults and inducing an inflammatory response if such insults are indeed detected. NLRP3 inflammasome can be stimulated by several factors at the level of inflammasome assembly activation. Those factors include potassium efflux from intracellular fluids, reactive oxygen species (ROS), mitochondrial and phagolysosomal damage, as well as pathogens, such as bacteria, viruses, fungi and parasites (He et al., 2016; Zheng et al., 2020). Overexpression of active caspase 1 in microglial cells has been found in patients suffering from mild cognitive impairment or AD (Heneka et al., 2013). In addition, NLRP3 receptor expression is also transcriptionally controlled by NF-κB (Bauernfeind et al., 2009). It has been recently found that fibrillary A*β* aggregates can directly activate NLRP3 inflammasomes in microglial cells, which promotes caspase 1 activation (Nakanishi et al., 2018; Lučiūnaitė et al., 2020). Moreover, it has been confirmed that NLRP3 inflammasome indeed contributes to A*β* deposits formation in mice (Venegas and Heneka, 2019). In physiology, IL-1*β* can increase core body temperature through stimulation of thermoregulation center in the hypothalamus. In addition, IL-1*β* can promote sleep and sickness behavior in response to infections. While small amounts of IL-1*β* can promote long term potentiation (LTP) and thus acquisition of cognitive skills, large amounts of IL-1*β* are thought to be detrimental in the course of AD, mainly through suppression of hippocampal neurogenesis (Hevett et al., 2012).

Inflammasome activation in mouse microglial cells has been found to promote formation of neurofibrillary tangles (Ising et al., 2019). This phenomenon is mediated by increased phosphorylation of tau proteins by p38 kinase, stimulated by IL-1 (Li et al., 2003). In addition, pro-inflammatory stimuli, such as lipopolysaccharide (LPS) have also been found to promote hyperphosphorylation of tau proteins by cyclin-dependent kinase 5 (CDK-5), which can in turn be stimulated by IL-6 (Quintanilla et al., 2004; Kitazawa et al., 2005).

**FIGURE 1:**
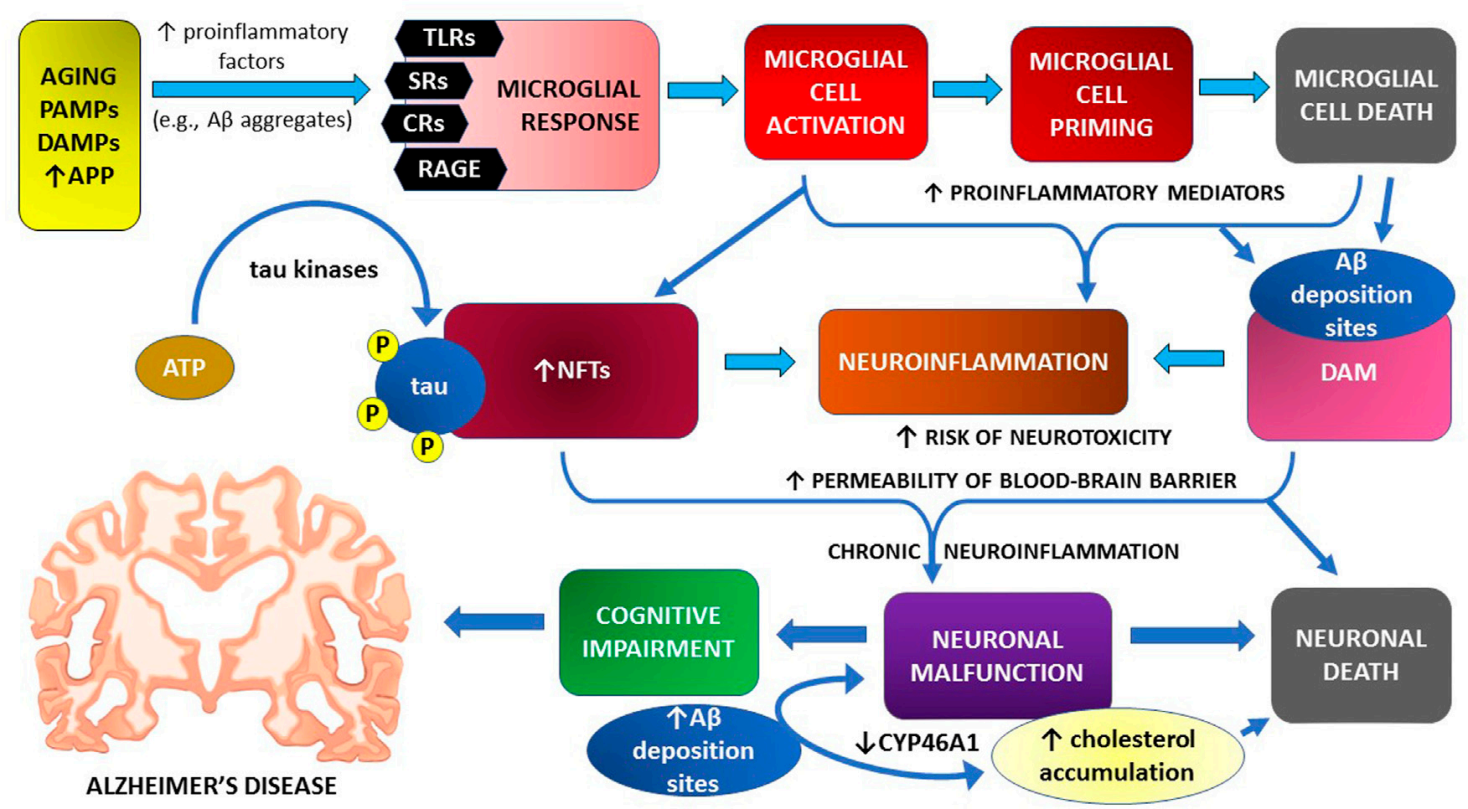
Microglial cell response and neuroinflammation in the pathomechanism of cognitive impairment—the main symptom of Alzheimer’s disease.

Mechanisms of microglial cell contribution to neuroinflammation and cognitive impairment in the course of Alzheimer’s disease are graphically illustrated in Figure 1

### Sirtuin functions and their expression in the CNS

Sirtuins comprise a family of evolutionarily conserved enzymes performing NAD^+^ dependent protein deacetylation/deacylation (North and Verdin, 2004). Sirtuins have been initially discovered as transcription silencing factors in yeast, extending yeast replicative lifespan through histone deacetylation, resulting in heterochromatin formation and silencing mating-associated genes (Kaeberlein et al., 1999; Imai et al., 2000). More recently, sirtuins have been found to extend lifespan, or at least healthspan, in flatworms, fruit flies and mice (Imai and Guarente, 2016). In addition, sirtuin induction has been associated with caloric restriction-dependent lifespan extension in mammals (Bishop and Guarente, 2007; Guarente, 2013). Seven sirtuins have been identified in mammals so far; they can possess various enzymatic activity profiles and different subcellular location, but all of them share evolutionarily conserved catalytic core, consisting of NAD^+^ binding domain and zinc binding domain. Sirtuin domains other than aforementioned catalytic core seem to take part in substrate recognition and activity regulation (Feldman et al., 2012). Sirtuins can deacetylate both histone and non-histone substrates, including transcription factors, manganese superoxide dismutase (MnSOD) and tubulin. Mammalian sirtuins (SIRT1—7) have different profiles of action, substrate affinity, and subcellular compartmentation. Yet, all of them share a similar catalytic domain and use NAD^+^ as a co-substrate. Although initially identified as deacetylases, sirtuins are now known to have much more kinds of enzymatic activity, including deacylase and O-ADP-ribosylase activity (Michan and Sinclair, 2007). SIRT3, SIRT4, and SIRT5 are mitochondrial proteins, while SIRT1, SIRT6 and SIRT7 are nuclear enzymes, and—as such—can take part in the epigenetic regulation of cell phenotype, especially that they target histones and transcription factors. SIRT2 can be shuttled between nucleus and cytoplasm, depending on the phase of the cell cycle. Through exerting posttranslational regulatory modifications (PTRMs) on their target substrates, sirtuins can regulate a plethora of intracellular processes, such as energy expenditure, metabolic pattern, ROS concentration, DNA conservation, DNA damage repair, and cellular aging (Michan and Sinclair, 2007; Haigis and Sinclair, 2010; Houtkooper et al., 2012).

Sirtuins are quite abundantly expressed in the brain (Sidorova-Darmos et al., 2014; Jayasena et al., 2016). There is much evidence that various sirtuins are produced in different regions of the brain, while their activity can change with age. Furthermore, sometimes sirtuins’ enzymatic activity becomes reduced with age, despite their concentration increasing, which has been confirmed in mice in reference to SIRT1 and is generally attributed to falling concentration of NAD^+^ within cells (Braidy et al., 2015). However, in some circumstances, e.g., in rat hippocampal cells, SIRT1 expression becomes also reduced with age (Yan et al., 2019). The same problem may exist in reference to mitochondrial sirtuins (SIRT3-5) (Braidy et al., 2015). In neurons and glial cells cultured *in vitro*, the most expressed sirtuins include SIRT1-3 (Jayasena et al., 2016). Moreover, the levels of SIRT1 and SIRT3 in AD patients brains are reduced (Lutz et al., 2014; Lee et al., 2018; Yin et al., 2018). In addition, even in the plasma obtained from old mammals, SIRT1 and SIRT3 concentrations are decreased, which is correlated with general frailty (Kumar et al., 2014). In AD patients serum levels of SIRT1 are reduced, while SIRT6 levels are also reduced—both in the CNS and in the plasma, both in AD patients and in mouse models of AD (Kumar et al., 2013; Jung et al., 2016; Kaluski et al., 2017).

#### The role of sirtuins in maintenance of brain homeostasis and prevention of Alzheimer’s disease

Sirtuins play an important role in the maintenance of neuronal well-being during aging (Herskovits and Guarente, 2014). In addition, they regulate many AD-associated processes, including APP processing, tau protein processing, mitochondrial functions, oxidative stress level, and neuroinflammation (Lalla and Donmez, 2013; Jęśko et al., 2017; Lee et al., 2018; Mohamad Nasir et al., 2018; Rizzi and Roriz-Cruz, 2018).

#### Sirtuin actions inhibiting A*β* aggregate formation and promoting their degradation

SIRT1 inhibits A*β* aggregate production through activating a disintegrin and metalloproteinase domain-containing protein 10 (ADAM-10), and thus stimulating APP processing to non-amyloidogenic, soluble metabolites, called soluble APP*α* (sAPP*α*) (Qin et al., 2006; Lee et al., 2014; Zhang et al., 2020). Furthermore, SIRT1 facilitates A*β* peptide degradation by upregulating lysosome number in primary astrocytes (Li et al., 2018).

**FIGURE 2:**
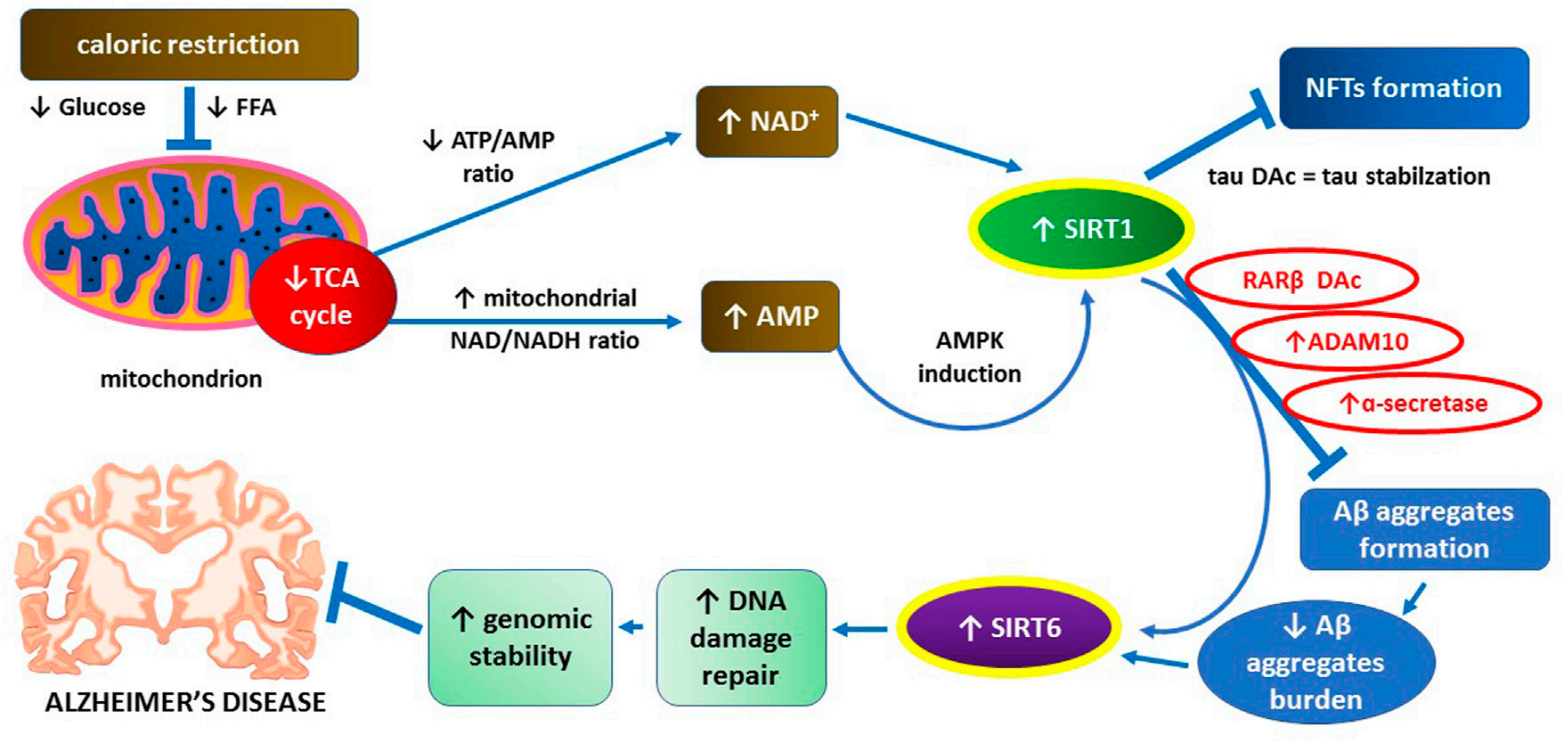
The key role of SIRT1 in supporting neuroprotective action of SIRT6 through preventing A*β* aggregates formation.

SIRT1 levels in cerebral cortex of AD patients are reduced, and decreased SIRT1 concentration and activity are positively correlated with A*β* deposits formation in the extracellular space and NFT formation inside neurons (Julien et al., 2009). Furthermore, caloric restriction as a classic SIRT1 inducer alleviates A*β*-dependent pathology on animal models of AD (Wang et al., 2005; Qin et al., 2006). A*β* aggregates can reduce the expression of SIRT6 which is important for DNA damage repair and maintenance of genomic stability (Kugel and Mostoslavsky, 2014; Jung et al., 2016). Increased expression of SIRT6 may protect hippocampal neurons from A*β*-dependent DNA damage (Jung et al., 2016). Main mechanisms underlying inhibitory actions of SIRT1 towards A*β* aggregate deposition and related pathology are graphically illustrated in Figure 2.

#### Sirtuin actions inhibiting NFT formation through preventing hyperphosphorylation of tau proteins

NFT formation is usually preceded by increased posttranslational regulatory modifications of tau proteins, such as phosphorylation and acetylation (Wang and Mandelkow, 2016; Guo et al., 2017). Acetylation of tau proteins inhibits their degradation, especially in reference to their phosphorylated forms, which promotes tau accumulation and neurotoxicity (Min et al., 2010; Cohen et al., 2011; Min et al., 2015; Tracy et al., 2016). In mouse models of tauopathy, SIRT1 overexpression or activation counteracts tau acetylation, which alleviates tau-related neurotoxicity (Min et al., 2018). It has been also shown that tau acetylation in mice can be promoted by A*β* aggregates through inhibition of SIRT3 expression (Yin et al., 2018). In mouse hippocampal neurons, SIRT3 activity induction reduces the extent of tau acetylation, while SIRT3 inhibition has the opposite effect (Li et al., 2019). Phosphorylation of tau proteins may be prevented by SIRT6 which inhibits glycogen synthase kinase 3 (GSK3) as a tau-phosphorylating enzyme (Kaluski et al., 2017).

**FIGURE 3:**
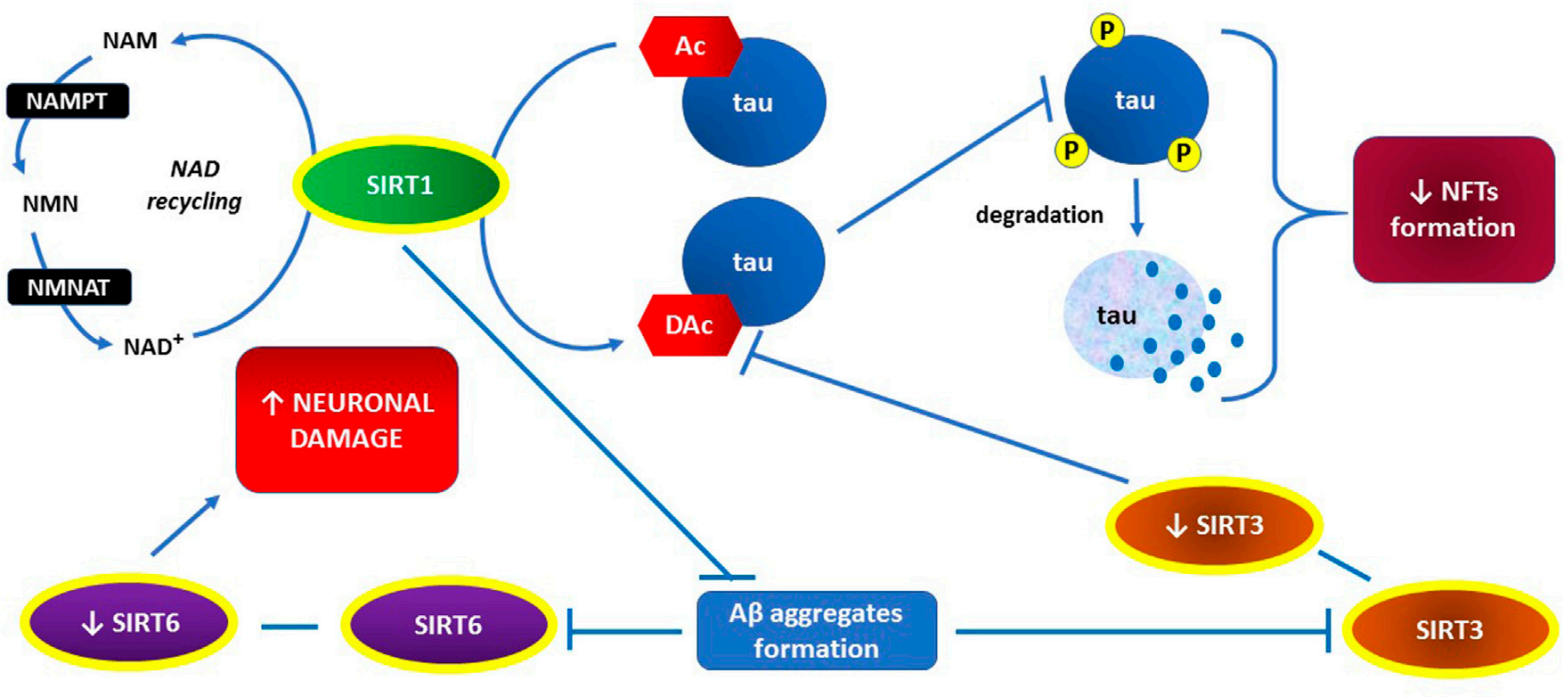
The key role of SIRT1 in both, inhibiting NFTs formation through preventing hyperphosphorylation of tau proteins and recovering activities of SIRT3 and SIRT6 through preventing A*β* aggregates formation.

The key actions of sirtuins, preventing NFT formation, are presented graphically in Figure 3.

### Anti-neuroinflammatory actions of sirtuins, through inactivation of p65 subunit of NF-κB

NF-κB activation may occur through the canonical or non-canonical pathway. Still, in standard conditions, the canonical pathway is blocked by default due to IkB proteins, which sequestrate NF-κB in the cytoplasm. However, pro-inflammatory stimuli may activate IkB kinase (IKK), which promotes IkB degradation through inhibitory phosphorylation, and thus relocation of NF-κB to the cell nucleus. Sirtuins may inhibit NF-κB both directly and indirectly. Firstly—SIRT1 and SIRT2 can deacetylate NF-κB’s p65 subunit at lysine 310, which directly inhibits NF-κB activity (Yeung et al., 2004). Furthermore, such acetylation impedes methylation of adjacent lysine residues (K314 and K315), promoting ubiquitination and degradation of p65 (Rothgiesser et al., 2010; Yang et al., 2010). Secondly—SIRT1 can inhibit NF-κB through inhibitory phosphorylation of its transcriptional activators, such as PARP-1 and p300 histone acetyltransferase (Bouras et al., 2005; Rajamohan et al., 2009). Thirdly—SIRT1 and SIRT6 may inhibit the expression of NF-κB target genes due to transcriptional silencing through H3K9 DAC (Kawahara et al., 2009). In this way, SIRT1 exerts anti-inflammatory actions, counteracting neuroinflammation. A*β* interactions with microglial cells promote p65 subunit acetylation, while SIRT1 activation or overexpression prevents this effect. Therefore, SIRT1 protects CNS from A*β* neurotoxicity through inhibiting NF-κB dependent pro-inflammatory signaling pathway (Chen et al., 2005; Yang et al., 2012).

SIRT6 can induce the production of IkB at the level of transcription, which exerts an anti-inflammatory effect because IkB blocks the canonical pathway of NF-κB activation by default (Kawahara et al., 2009). In addition, SIRT6 may both desensitize cells to TNF-alpha, an upstream inducer of NF-κB, and inhibit TNF-alpha secretion. SIRT1 and SIRT6 actions described above are primarily responsible for their anti-inflammatory effects.

In addition, SIRT1 inhibits the production of IL-1*β*, a pro-inflammatory cytokine. This effect is dependent on activatory deacetylation of DNA (cytosine-5)-methyltransferase 1 (DNMT1)—an enzyme that inhibits biosynthesis of IL-1*β* at the level of transcription, through DNA methylation at IL-1*β* proximal promoter (Peng et al., 2011; Cho et al., 2015; Heo et al., 2017). If SIRT1 activity is reduced with age, the extent of DNA methylation at IL-1*β* proximal promoter is also reduced, which can facilitate IL-1*β* biosynthesis at the level of transcription, thus aggravating neuroinflammation. SIRT1 activators, such as resveratrol, can prevent this effect (Yan et al., 2019).

SIRT2 can also inhibit neuroinflammation through direct deacetylation of p65 at lysine 310 (Rothgiesser et al., 2010; Pais et al., 2013). SIRT2 inhibition may promote transition of microglial cells from homeostatic/quiescent phenotype to pro-inflammatory phenotype on mouse model of traumatic brain damage, through reactivation of NF-κB dependent pro-inflammatory signaling pathway (Yuan et al., 2016). It has been also found that SIRT2 overexpression in rats reduces neuroinflammation exactly through p65 deacetylation (Zhang and Chi, 2018). On the other hand, results of other research studies reveal some potentially pro-inflammatory actions of SIRT2. Inhibition of SIRT2 blocks NF-κB molecule translocation to cell nucleus, thus abrogating TNF-α and IL-6 expression in mouse microglial cells exposed to LPS. Thus, SIRT2 seems to be necessary to induce LPS-dependent neuroinflammation (Wang et al., 2016). Pharmacologic inhibition of SIRT2 reduces TNF-*α* and nitric oxide production in LPS-exposed microglial cells (Harrison et al., 2018). Furthermore, SIRT2 inhibition attenuates α-synuclein neurotoxicity on mouse models of Parkinson’s disease (Outeiro et al., 2007; Chen et al., 2015). Similarly, SIRT2 inhibition in mice alleviates cognitive deficits on mouse models of Alzheimer’s disease, through inhibition of A*β* formation (Biella et al., 2016). Although TNF-*α* signaling dependent on TNF-R_1_ receptor is thought to be pro-inflammatory and thus deleterious in the course of AD, TNF-α may also exert some neuroprotective effects through acting on TNF-R_2_ receptors. Since neuroprotective actions of TNF-*α* may include protection against demyelination, excitotoxicity and cerebral ischemia (Probert, 2015), this may—at least in part—explain why SIRT2 inhibition can be neuroprotective in some circumstances. Therefore, further research studies are needed to verify overall effect of SIRT2 and its inhibitors towards neuroinflammation in the course of AD, although inhibitors of TNF-*α* dependent signaling usually improve the cognitive performance of AD patients (He et al., 2007). In general, the outcome of NF-κB activation depends very much on the cell type and the stimuli present, since it determines which signaling pathway becomes activated. This may account for some discrepancies related to SIRT2 activation/inhibition effects towards inflammatory response.

Unlike TNF-α, IL-6 seems to have mainly deleterious effects towards aging brain, through promoting gliosis and inflammation, inhibiting LTP in hippocampal neurons, enhancing the neurotoxic properties of NMDA, as well as reducing adult neurogenesis in the hippocampal dentate gyrus (Godbout and Johnson, 2004). Furthermore, severity of dementia in the course of AD is positively correlated with IL-6 concentration in serum (Kalman et al., 1997). When having taken into consideration that IL-6 production is stimulated by NF-κB and its upstream inducers, both SIRT1 and SIRT6, which inactivate NF-κB, may exert their beneficial effects on the brain exactly through possessing this property.

**FIGURE 4:**
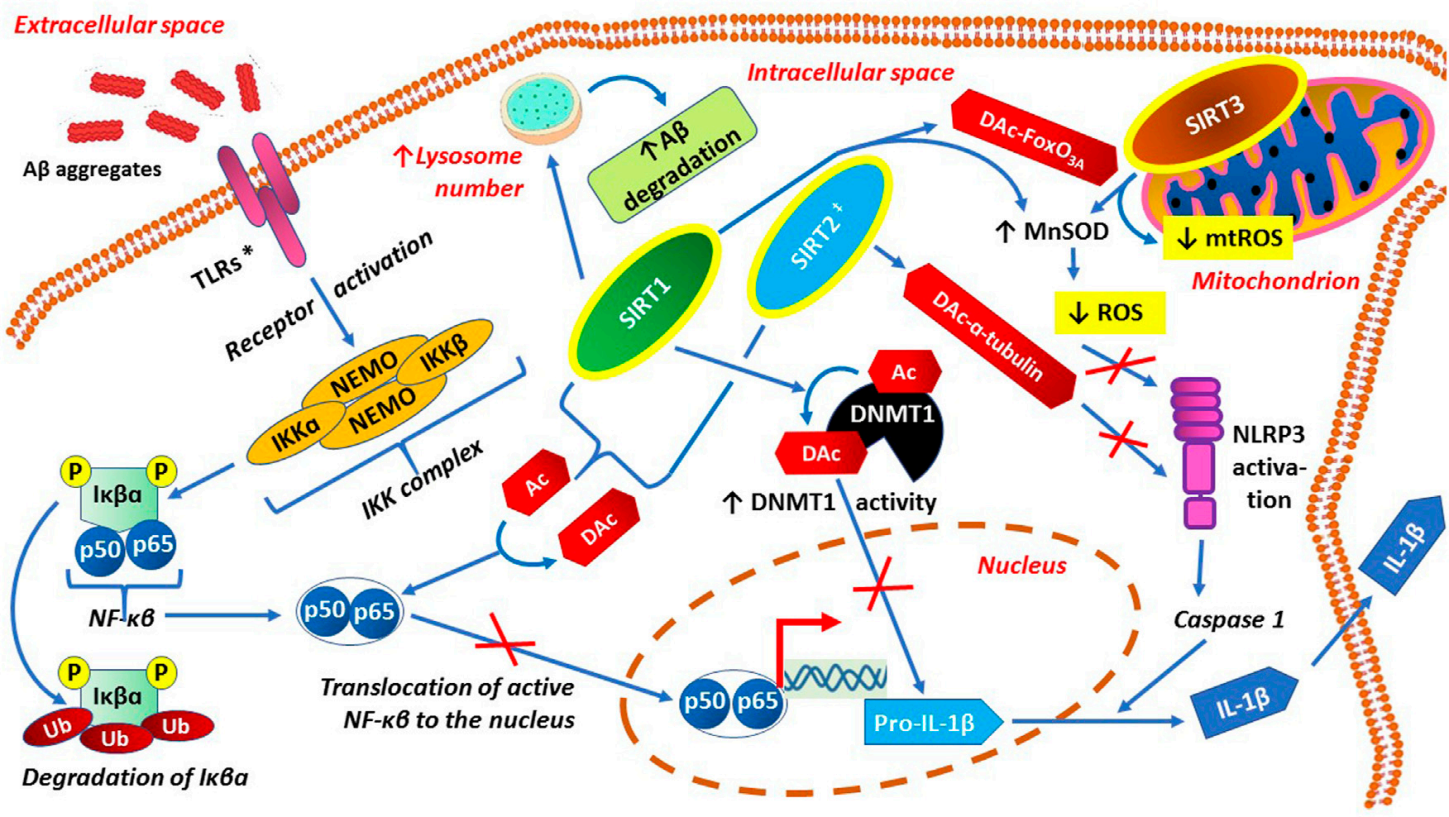
Anti-neuroinflammatory actions of sirtuins, through inactivation of p65 subunit of NF-κB, activation of DNMT1 and anti-oxidative effects.

Main preventive actions of sirtuins against both neuroinflammation and neuroinflammation-related oxidative stress are presented graphically in Figure 4.

### Sirtuin actions inhibiting neuroinflammation and neuronal death through their anti-oxidative effects

SIRT3, a mitochondrial sirtuin, is quite significant for counteracting oxidative stress, since it both optimizes the action of respiratory chain enzymes and activates MnSOD. Therefore, SIRT3 inhibits ROS production and facilitates ROS inactivation (Ansari et al., 2017; Meng et al., 2019). SIRT3 activity falls during neuroinflammation in LPS-exposed microglial cells, while SIRT3 activation can prevent both LPS-induced neuroinflammation and mitochondrial dysfunction resulting in microglial cell death (Zhou and Jiang, 2019). Microglia-derived pro-inflammatory cytokines may induce apoptosis of neural tissue stem cells, as well as inhibit their proliferation. Using co-cultures of microglial cells and neural tissue stem cells, it has been found that A*β*-induced microglial cell transition to pro-inflammatory phenotype results in neural tissue stem cell necrosis through cytokine-dependent inhibition of SIRT3 and MnSOD, with a subsequent rise in intracellular ROS concentration. SIRT3 activation or overexpression protects the cells from such cytokine-dependent oxidative stress (Jiang et al., 2017). SIRT3 also protects mice from cognitive deficits induced by surgery/anesthesia brain injury. In old mice with cognitive impairments, loss of function of both SIRT3 and MnSOD has been found in hippocampal cells (Liu et al., 2021).

SIRT1 can also counteract oxidative stress through forkhead box O_3A_ (FoxO_3A_) deacetylation, resulting in MnSOD activation by deacetylated FoxO_3A_ (Brunet et al., 2004). As to SIRT3, it may promote both FoxO_3A_ deacetylation and direct activation of MnSOD, also through deacetylation (Tao et al., 2014; Rangarajan et al., 2015).

Both ROS and mitochondrial degradation products can exert pro-inflammatory actions through activating NLRP3 inflammasome (Zhou et al., 2011; Wilkins et al., 2017). In this context, hyperactivation of inflammasomes as innate immunity components may promote neuroinflammation in the course of AD, while inflammasome activity inhibition can prevent neuroinflammation (Venegas and Heneka, 2019). This is why both SIRT1 and SIRT3 can prevent neuroinflammation through their mitochondria-protective and antioxidative effects (Zhang et al., 2017; Zou et al., 2018). SIRT2 may also inhibit NLRP3 inflammasome through deacetylation of α-tubulin, which is necessary in its acetylated form for inflammasome activation (Misawa et al., 2015). SIRT2 may also directly deacetylate pyrin domains significant for inflammasome activation (He et al., 2020).

Growing evidence suggests that mitochondrial dysfunction within CNS cells, as well as the resulting oxidative stress, are strongly associated with Alzheimer’s disease (Manoharan et al., 2016; Kausar et al., 2018; Llanos-González et al., 2020). In this context, activation of SIRT1 and SIRT3 can prevent AD through boosting their antioxidative and mitochondria-protective actions (Woodbury et al., 2013; Ye et al., 2019).

A*β* aggregates can also activate NLRP3 inflammasome through inducing phagolysomal damage in microglial cells, followed by leakage of lysosomal proteases and cathepsin B into the cytoplasm (Halle et al., 2008; Heid et al., 2013; Wu et al., 2013; Campden and Zhang, 2019; Kelley et al., 2019). Since SIRT1 inhibits A*β* aggregate formation, increasing its activity in the brain may causally prevent AD-associated pathology (Gay et al., 2020).

Conclusion: boosting sirtuins activity, especially in reference to SIRT1 and SIRT3, both through allosteric activation and through NAD^+^ replenishment, can be regarded as very promising strategy of promoting brain homeostasis and AD prevention, especially if the applied boosters are well-tolerated, safe, and easily crossing the blood-brain barrier.

## Discussion

Some pathomechanisms of Alzheimer’s disease have not been addressed in this article. Those mechanisms not addressed include: pathogenic role of some bacteria, such as *P. gingivalis*, in the induction of neuroinflammation (Dominy et al., 2019), as well as potentially pathogenic role of some metals—especially aluminum—in promoting A*β* aggregate oligomerization (Zhang et al., 2019). Similarly, we have not discussed the detailed mechanisms of neurotoxicity of misfolded proteins, such as tau proteins. In spite of that, it can be assumed that neuroinflammation etiology does not take part in the mechanisms of neuroinflammation-alleviating action of sirtuins. In other words, sirtuins can alleviate neuroinflammation regardless of its cause, since they inhibit an essential pro-inflammatory signaling pathway dependent on NF-κB. Although eradication of pro-neuroinflammatory bacterial infections is useful and desired, detailed microbiology of those infections is not a topic of this paper, while AD-promoting effect of such infections may occur either through stimulated production of pro-inflammatory cytokines in some regions adjacent to the CNS, so that those pro-inflammatory cytokines can act on the CNS in a paracrine manner, or through penetration of some bacterial toxins through the blood-brain barrier, thus exerting a direct neurotoxic effect.

As to the role of aluminum in the pathogenesis of Alzheimer’s disease, it seems to basically consist in promoting the oligomerization of peptides produced from APP by *β*- and *γ*-secretases, which raises the risk of A*β* aggregate formation. Since SIRT1 induction stimulates *α*-secretases, thus reducing the risk of APP processing by *β*- and *γ*-secretases, it can neutralize aluminum influence on the CNS, because aluminum excess seems to be harmful only if there is an already existing excess of *β*- and γ-secretase products.

Another matter is *de facto* lack of empirical, measurable effects of applying sirtuin boosters discovered so far on the course of Alzheimer’s disease in hitherto performed clinical trials. However, it should be taken into consideration that sirtuin allosteric activators discovered so far do not cross blood-brain barrier with 100% efficacy, while sirtuin activity boosters in the form of close NAD^+^ precursors are not widely available in pharmacy retail trade—either as medications or as dietary supplements, which limits their use. In addition, it is worth remembering that beneficial effects of sirtuins towards the course of Alzheimer’s disease, discussed in this paper, are mainly preventive, which means that empirical and measurable confirmation of the sirtuins’ actions assumed may require introducing the treatment with sirtuin activity boosters 15–20 years prior to the onset of AD clinical symptoms, in case of detection of AD risk factors (e.g., A*β* deposits or A*β*-associated alterations in neuroimaging). Moreover, hitherto known allosteric activators of sirtuins, such as resveratrol (SIRT1 activator), honokiol (SIRT3 activator), or SRT1720 may require chemical modifications to improve their crossing through blood-brain barrier, while sirtuin activity boosters in the form of close NAD^+^ precursors require introducing to the pharmaceutical retail market to provide their broad availability for people who would like to use them within the frames of AD prevention. Summing up: even if sirtuin boosters as a possible method of AD prevention were introduced today, their beneficial effects might be observed in a time interval equivalent to the time amount usually required for a progression of AD from its initial pathological and molecular manifestations to its clinical stage.

Someone could ask whether a preventive action of sirtuin activation refers to all sirtuins, or merely to those widely described as neuroprotective. The answer is: probably such a beneficial effects refer to all sirtuins, with only SIRT2 being a possible exception, although even this is uncertain, since there are some results of research studies indicating anti-inflammatory actions of SIRT2 (Rothgiesser et al., 2010; Pais et al., 2013; Yuan et al., 2016).

Another possible question is whether sirtuins are the only enzymes using NAD^+^ as a coenzyme/co-substrate. According to current knowledge, the answer is “no”, because there are strong premises to claim that beneficial effects of NAD^+^ replenishment are strictly correlated exactly with boosting the activity of sirtuins (Imai and Guarente, 2016), while focusing on all enzymes using NAD^+^ as a coenzyme would largely exceed the scope of this paper.

The question related to the previous one is whether using close metabolic precursors of NAD^+^ affects the activity of enzymes other than sirtuins. The answer is “yes”, and thus it cannot be excluded that at least some beneficial effects of NAD^+^ replenishment strategies are mediated by affecting enzymes other than sirtuins (e.g., poly-ADP-ribosyltransferases, glycohydrolases, mitochondrial enzymes coupling TCA reactions with oxidative phosphorylation and ATP biosynthesis). In other words, it is possible that NAD^+^ replenishment exerts its beneficial effects through affecting the activity of enzymes other than sirtuins. Although there are some premises that NAD^+^ precursors exert their beneficial effects indeed through sirtuin activation (Guarente, 2013; Imai and Guarente, 2016), additional research studies should be made to pinpoint the mechanisms of action of NAD^+^ replenishment strategies by comparing the phenotypic beneficial effects related to NAD^+^ replenishment with the effects achieved through selective overexpression of particular sirtuins. This kind of research studies may be necessary to verify whether NAD^+^ replenishment effects are sirtuin-specific or not.
