# Supplementary material for: Sirtuins promote brain homeostasis, preventing Alzheimer’s disease through targeting neuroinflammation
Source: Front Physiol. 2022 Aug 15;13:962769. doi: 10.3389/fphys.2022.962769 (PMC9420839; doi:10.3389/fphys.2022.962769)
Supplement: Supplementary file 1 [file DataSheet1.docx]

**CAPTIONS TO THE FIGURES**

**Figure 1.** Microglial cell response and neuroinflammation in the pathomechanism of cognitive impairment – the main symptom of Alzheimer’s disease.

Aging, PAMPs, DAMPs, as well as other conditions with accumulation of APP can predispose to Aβ aggregates formation with subsequent microglial cell priming and activation. And this in turn may promote neuroinflammation – both through increased synthesis of pro-inflammatory cytokines and through inducing biosynthesis of other pro-inflammatory cytokines in astrocytes. The microglial response results from the stimulation of various receptors including TLRs, SRs, CRs, and RAGE. A positive correlation has been observed between microglial cell response (activation, priming, and death) and neuroinflammation, and cognitive impairments. In the regions adjacent to Aβ deposition sites, DAM can be found. Ineffective phagocytosis of Aβ by microglial cells can result in their necrosis, followed by Aβ release back to the extracellular space and aggravating neuroinflammation. In addition, hyperactivation of microglial cells can promote NFTs formation through hyperphosphorylation of tau proteins inside neurons. Several neurodegenerative diseases and changes in cognition are related to altered CYP46A1 expression, an enzyme that is responsible for brain cholesterol elimination and therefore plays a crucial role in the control of brain cholesterol homeostasis.

*Aβ aggregates – aggregates of amyloid-beta; APP – amyloid-precursor protein; ATP – adenosine triphosphate; CRs – complement receptors; CYP46A1 – cholesterol 24-hydroxylase; DAM – disease-associated microglia; DAMPs – damage-associated molecular patterns ; NFTs – neurofibrillary tangles; P – phosphorylation; PAMPs – pathogen-associated molecular patterns; RAGE – receptor for advanced glycation end products; SRs – scavenger receptors; TLRs – toll-like receptors;*

**Figure 2.**

The key role of SIRT1 in supporting neuroprotective action of SIRT6 through preventing Aβ aggregates formation.

SIRT1 can inhibit formation of both NFTs and Aβ. Deacetylation of tau proteins by SIRT1 leads to tau stabilization, preventing NFTs formation. Inhibition of Aβ aggregates formation involves deacetylation of RARβ by SIRT1. Thus, SIRT1 directly activates the transcription of the gene encoding the α-secretase, ADAM10. Such activation of α-secretase prevents amyloidogenesis. Moreover, by reduction of Aβ aggregates burden SIRT1 “rescuing” SIRT6 activity. Increased expression of SIRT6 may protect neurons from Aβ-dependent DNA damage in Alzheimer’s disease. Caloric restriction is helpful to restore optimal SIRT1 activity.

*Aβ aggregates – aggregates of amyloid-beta; ADAM10 – a disintegrin and metalloproteinase domain-containing protein 10; AMP – adenosine monophosphate; AMPK – 5′-adenosine monophosphate (AMP)–activated protein kinase; ATP – adenosine triphosphate; DAc – deacetylation; FFA – free fatty acids; NAD – nicotinamide adenine dinucleotide; NADH – reduced nicotinamide adenine dinucleotide; NFTs – neurofibrillary tangles; RARβ – retinoic acid receptor β; SIRT1, 6 – sirtuins: 1 and 6; TCA – the tricarboxylic acid cycle or the Krebs cycle*

**Figure 3.** The key role of SIRT1 in both, inhibiting NFTs formation through preventing hyperphosphorylation of tau proteins and recovering activities of SIRT3 and SIRT6 through preventing Aβ aggregates formation.

SIRT1 deacetylates tau protein, which inhibits its hyperphosphorylation and facilitates degradation, and thus inhibits NFTs formation. Aβ aggregates produce SIRT3 inhibition with subsequent inhibition of an alternative pathway of tau deacetylation. Aβ aggregates inhibit SIRT6 activity promoting neuronal damage, but SIRT1 restores activity of SIRT6 by limiting formation of Aβ aggregates. SIRT1 as NAD-dependent protein deacetylase is regulated by nicotinamide, which is recycled to NAD through NAMPT and NMNAT.

*Aβ aggregates – aggregates of amyloid-beta; Ac – acetylation; DAc – deacetylation; NAD – nicotinamide adenine dinucleotide; NAM – nicotinamide; NAMPT – nicotinamide phosphoribosyltransferase; NFTs – neurofibrillary tangles; NMN – nicotinamide mononucleotide; NMNAT – nicotinamide mononucleotide adenylyl transferease; P – phosphorylation; SIRT1, 3, 6 – sirtuins: 1, 3, and 6*

**Figure 4.** Anti-neuroinflammatory actions of sirtuins, through inactivation of p65 subunit of NF-κB, activation of DNMT1 and anti-oxidative effects.

Aβ aggregates activate cell membrane receptors that signaling though the NF-κB signaling pathway. SIRT1 exerts neuroprotective and anti-inflammatory effects, inhibiting both Aβ production and pro-inflammatory activation of microglial cells through abrogating NF-κB and IL-1β dependent signaling pathways . Both SIRT1 and SIRT2 can inhibit neuroinflammation via direct deacetylation of p65. SIRT1 and mitochondrial SIRT3 counteracts oxidative stress through FoxO_3A_ deacetylation, resulting in MnSOD activation and/or direct MnSOD activation via deacetylation. Thus limited formation of ROS inhibits NLRP3 activation. SIRT2 may inhibit NLRP3 through deacetylation of ɑ-tubulin. SIRT1 facilitates Aβ degradation by upregulating lysosome number [**XX**].

* TLRs are one example of a group of receptors that may be replaced with SRs or RAGE

**^‡^** SIRT2 actions seem to be more complex because on some research models, its inhibition abrogates neuroinflammation more effectively than its activation

*Li MZ, Zheng LJ, Shen J, et al. SIRT1 facilitates* *amyloid beta peptide degradation by upregulating lysosome number in primary astrocytes. Neural Regen Res. 2018;13(11):2005-2013. doi:10.4103/1673-5374.239449*

*Aβ aggregates – aggregates of amyloid-beta; Ac – acetylation; DAc* *– deacetylation; DNMT1 –* *DNA Methyltransferase 1; FoxO3A – the transcription factor Forkhead box protein O3a; IKKɑ – Inhibitor of nuclear factor kappa-B kinase subunit alpha; IKKβ – Inhibitor of nuclear factor kappa-B kinase subunit beta; IL-1β – interleukin 1 beta; MnSOD – manganese superoxide dismutase; mtROS – mitochondrial reactive oxygen species; NEMO – NF-kappa-βessential modulator NEMO also known as inhibitor of nuclear factor kappa-B kinase subunit gamma (IKK-γ); NF-κβ – nuclear factor kappa-light-chain-enhancer of activated β cells; P – phosphorylation; p50 – subunit p50 of the NF-kappaB p50/p65 heterodimer; p65 – subunit p65 of the NF-kappaB p50/p65 heterodimer; ROS – reactive oxygen species; SIRT1, 2, 3 – sirtuins: 1, 2, and 3; TLRs –* *Toll-like receptors; Ub – ubiquitination*
